# Perceptions of AI engaging in human expression

**DOI:** 10.1038/s41598-021-00426-z

**Published:** 2021-10-27

**Authors:** Alexander H. Bower, Mark Steyvers

**Affiliations:** Cognitive Sciences, University of California, Irvine, Irvine, 92697 USA

**Keywords:** Psychology, Human behaviour, Computational science

## Abstract

Though humans should defer to the superior judgement of AI in an increasing number of domains, certain biases prevent us from doing so. Understanding when and why these biases occur is a central challenge for human-computer interaction. One proposed source of such bias is task subjectivity. We test this hypothesis by having both real and purported AI engage in one of the most subjective expressions possible: Humor. Across two experiments, we address the following: Will people rate jokes as less funny if they believe an AI created them? When asked to rate jokes and guess their likeliest source, participants evaluate jokes that they attribute to humans as the funniest and those to AI as the least funny. However, when these same jokes are explicitly framed as either human or AI-created, there is no such difference in ratings. Our findings demonstrate that user attitudes toward AI are more malleable than once thought—even when they (seemingly) attempt the most fundamental of human expressions.

## Introduction

As the capabilities of artificial intelligence (AI) systems accelerate, they increasingly surpass us in several domains—even those once thought exclusively human. From making medical diagnoses^1,2^ to offering jail-or-release decisions^3^, algorithms can outperform human experts in a number of tasks. Given the capability of AI in such tasks, we as users should defer to their guidance to make optimal decisions. However, a growing body of evidence reveals certain biases in how users seek and weigh advice from algorithms compared to that of humans, resulting in sub-optimal decision making^4,5^. Given the prevalence of these systems and their demonstrated ability to inform high-stakes decisions, it is critical to identify and eliminate such biases.

While people have expressed skepticism toward algorithms for decades^6^, empirical work exploring these attitudes is quite recent. Such work has identified two primary forms of bias: *algorithm aversion*^7,8^ and *algorithm appreciation*^9^. Algorithm aversion occurs when users prefer human to algorithmic judgement—even when the latter is proven superior. Conversely, algorithm appreciation occurs when users prefer algorithmic to human judgment. These seemingly incompatible findings raise a host of important questions: Why does aversion occur in some contexts, while appreciation occurs in others? How strong are these biases? Can they be overcome (and, if so, how)?

These differences may be accounted for, in part, by task characteristics^5,7^. For instance, users tend to prefer human judgement on subjective tasks (e.g., book, movie, and joke recommendations^10,11^), while deferring to algorithmic judgement on objective tasks (e.g., logic problems^12,13^). However, it should be noted that users *have* demonstrated aversion to decision aids even when they are perfectly accurate in objective judgment (e.g., target detection)^14^ This suggests that users tend to discount advice from AI when a task invokes domains believed to be inextricably human, such as personal taste, intuition, and experience. We thus tested the hypothesis that these conditions elicit aversion through a task invoking a fundamentally subjective and human expression: Humor. Specifically, telling jokes.

Beyond meeting the criteria of subjectivity, this task is relevant for a number of reasons. First, humor is a universal phenomenon with which we all have experience and possess individual taste regarding^15^. Accordingly, people are able to make many short qualitative assessments of jokes in a single experimental session. Second, this topic is of interest to AI researchers, as developing embodied humor represents a critical challenge in naturalistic AI development^16^. Third, humorous virtual agents/AI have been shown to facilitate effective human-computer interaction^17^. One notable example is Morkes et al., who found that participants report more similarity to and cooperation with humorous agents when completing a task^18^. Lastly, it has been suggested that aversion occurs in part due to the belief that algorithms should perform with near-perfect accuracy^19^. This is presumably why users disproportionately punish mistakes when they are committed by an algorithm, compared to a human^8^. However, it is unclear if users will maintain this expectation in tasks lacking ground truth, such as joke-telling.

Indeed, individual and societal beliefs regarding AI’s comparative ability to produce humor remain largely unknown. The work conducted in this area has yielded interesting—if incomplete—results. For example, Tay et al. found that people perceive jokes as funnier when they are told by human actors than when they are told by robots, but only when joke content is non-disparaging^20^. Interestingly, they found that people express less disgust toward disparaging jokes when delivered by robot actors. Further supporting the importance of content and context in robot-delivered humor, Stoll et al. found that humor delivered by a robot is perceived as less appropriate in conflict mediation than when delivered by a fellow human^21^. It should be noted, however, that the physical embodiment of agents in these two studies almost certainly affected reception of their attempts at humor. Thus, the question largely remains: Do people respond differently to humor when it is believed to be created by an AI, versus a human?

## Results

Across two experiments, we address the following question: *Will people rate jokes as less funny if they believe an AI created them?* According to evidence showing that task subjectivity facilitates aversion, we hypothesized that they will. We tackled this question in two ways. In Experiment 1, we had participants rate the funniness of jokes and guess their likeliest source—a human or AI. We left these jokes’ actual sources ambiguous, forcing participants to make their own attributions. This allowed us to evaluate systematic differences in attribution between jokes considered low and high-quality. In Experiment 2, a new set of participants rated these same jokes. However, jokes were now explicitly labeled as either human or AI-created. This allowed us to assess potential differences in evaluations when jokes’ (purported) sources were transparent. If aversion is present, we should expect a systematic downgrading of jokes believed to be AI-created in each experiment, regardless of their actual source.

### Experiment 1

**Figure 1:**
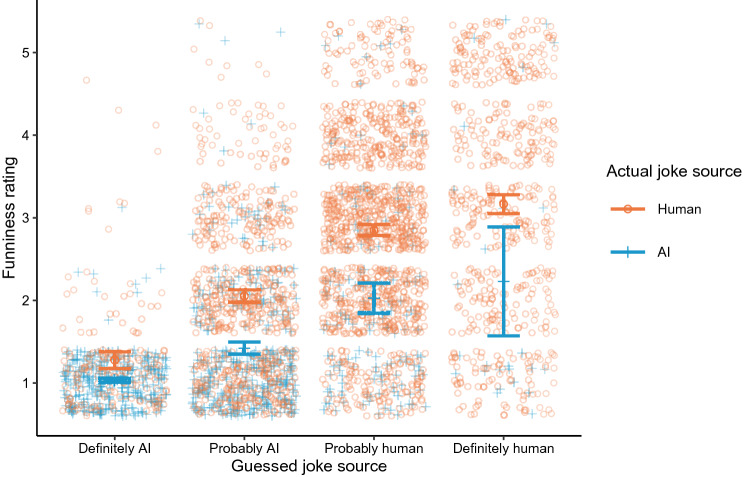
Ratings for human and AI-created jokes across their guessed sources. Error bars indicate 95% confidence intervals in rated funniness across actual joke sources (human or AI). Points represent an individual joke evaluation (rating and guessed/actual source). This figure was generated using R version 4.0.2 (https://www.r-project.org/).

In Experiment 1, participants were given a randomized sequence of jokes. Participants were not informed of these jokes’ actual sources. For each joke, they were asked to rate its funniness and to guess whether it was more likely created by a human or AI. Further details regarding the procedure can be found in the “Methods” section below. There are two main results, as shown in Table 1 and Fig. 1. First, participants guess that the funniest jokes were created by humans and that the least funny jokes were created by AI—regardless of their actual source. That is, funniness ratings increase as a function of how definitively human jokes are believed to be. Second, actual human-created jokes (*N* = 2580, *M* = 2.609, *SD* = 1.250) are rated funnier than actual AI-created jokes (*N* = 860, *M* = 1.416, *SD* = 0.829) across all guesses (*t*(2227.648) = $-\, 31.806$, *p* < 0.001, BF_10_> 100). These findings suggest that, in accordance with extant findings, humans prefer the output of other humans over AI in highly subjective tasks.

**Table 1:** Funniness ratings across actual and guessed joke sources.

Actual Source	Guessed Source	Mean	SD	N
AI	Definitely human	2.231	1.632	26
	Probably human	2.026	1.127	152
	Probably AI	1.422	0.732	379
	Definitely AI	1.033	0.197	303
Human	Definitely human	3.164	1.390	572
	Probably human	2.851	1.111	1171
	Probably AI	2.051	0.992	664
	Definitely AI	1.277	0.659	173

How accurate are participants at guessing jokes’ actual sources? More actual human-created jokes are correctly attributed to “probably” or “definitely” being human-created (*n* = 1743) than to being AI-created (*n* = 837), while more actual AI-created jokes are correctly attributed to being “probably” or “definitely” AI-created (*n* = 682) than to being human-created (*n* = 178). This indicates that participants are largely correct in their guesses.

We believe participants adhere to a reasonable heuristic here: When measuring the quality of jokes both by Reddit upvotes (see: “Data Availability”) and participant ratings, the funniest jokes *do* tend to be human-created. This rating behavior may reflect bias, but it also reveals accurate perceptions of the AI’s ability, as demonstrated by the high degree of accuracy in guesses. It is noteworthy, however, that many low-quality, human-created jokes are attributed to the AI (49% of jokes rated less than 3). This suggests that, even though low-quality jokes *are* largely AI-created, there is a tendency to attribute even low-quality human jokes to AI, further supporting the aversion hypothesis.

In sum, the findings from Experiment 1 demonstrate that when joke source is not provided, participants tend to attribute high-quality jokes to humans and low-quality jokes to AI. Further, while these attributions are quite accurate, evidence suggests a bias to attribute low-quality, human-created jokes to AI. These results provide a baseline for attitudes regarding AI’s ability to create jokes compared to humans and suggest the presence of algorithm aversion. However, will these attitudes persist when joke sources *are* provided? We tackled this question in Experiment 2.

### Experiment 2

In Experiment 2, a new set of participants rated the funniness of the same jokes from Experiment 1, again presented in randomized order. However, each joke was now explicitly framed (*i.e.*, labeled) as being either human or AI-created. The framing for each human-created joke was counterbalanced such that they were all equally-represented as human and AI-created. All AI-created jokes were accurately framed to improve the feasibility of the purported AI’s output.

**Figure 2:**
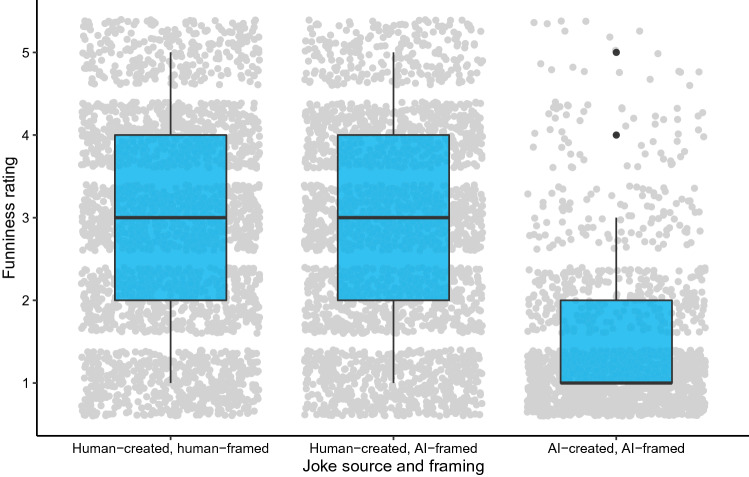
Funniness ratings for jokes across their actual source and framing. Dots indicate an individual joke rating under each framing. This figure was generated using R version 4.0.2 (https://www.r-project.org/).

The main finding is that there is no difference in funniness ratings for human-created jokes alternatively framed as human (*N* = 2490, *M* = 2.828, *SD* = 1.279) or AI-created (*N* = 2490, *M* = 2.765, *SD* = 1.274) (*t*(4978) = $-\, 1.732$, *p* $=$ 0.083, BF_10_ = 0.142), as shown in the left two data columns of Fig. 2. This indicates that, regardless of framing, participants rate human-created jokes equally. (Note that the Bayes factor shows not only that there is no significant difference, but that there is evidence that the funniness ratings are essentially the *same* between framings.) Critically, these results suggest that aversion is *not* present when participants are told the purported source of each joke. This result is inconsistent with the aversion hypothesis, as participants do not systematically downgrade jokes strictly because they are told that an AI created them.

In addition to this key finding, we observe other interesting results. First, human-created and framed jokes are rated significantly higher than their correctly AI-framed counterparts (*N* = 1660, *M* = 1.421, *SD* = 0.829) (*t*(4145.024) = $-\,42.987$, *p* < 0.001, BF_10_ > 100). Furthermore, human-created but AI-framed jokes are also rated higher than their correctly AI-framed counterparts (*t*(4145.756) = $-\,41.164$, *p* < 0.001, BF_10_ > 100). These results reinforce the fact that jokes are rated consistently based on quality, not purported origin.

Was our deception effective? Most participants (*n* = 68; 81.928%) report that they did *not* spot our deception. When asked to describe when and how they detected it, those who did cite the experimental design (e.g., “I don’t think there’s any reason to indicate “AI generated” or “human generated” unless you are looking for bias in responses to the jokes”), previous exposure to jokes (e.g., “I had heard some of the jokes that the AI supposedly wrote, years ago”), the disparity between misrepresented and accurately-framed AI joke quality (e.g., “When I noticed that some of the AI jokes were obviously more coherent than others”), and/or intuition about AI joke quality (e.g., “I just figured that AIs aren’t making up jokes especially really bad ones”). Our main result remains when these individuals are excluded from analysis. That is, for individuals who reported being deceived, there is still no difference in ratings between human-labeled (*N* = 2040, *M* = 2.756, *SD* = 1.269) and AI-labeled (*N* = 2040, *M* = 2.691, *SD* = 1.261) human-created jokes (*t*(4078) = $-\,1.646$, *p* = 0.100, BF_10_ = 0.136). Thus, the effectiveness of our cover story suggests a willingness to believe in AI’s ability to produce human-quality jokes.

Lastly, we compared results between experiments to verify consistency in joke ratings and to identify potential differences in rating trends. Mean joke funniness is higher in Experiment 2 than in Experiment 1 (*t*(10078) = 5.175, *p* < .001, BF_10_ > 100). However, only four jokes vary in pairwise magnitude across experiments, suggesting overall reliability in joke ratings.

Overall, the findings in Experiment 2 challenge the hypothesis that there is bias against human-created but AI-framed jokes. Thus, unlike in Experiment 1, there is no indication that algorithm aversion is present.

## Discussion

Across two experiments, we tested the hypothesis that task subjectivity invites algorithm aversion by examining whether people systematically downgrade ratings for jokes believed to be AI-created. The results from Experiment 1 show that people rate jokes guessed to be AI-created more harshly when no source is provided. Furthermore, they frequently attribute low-quality, human-created jokes to AI. This suggests that people may hold latent biases regarding AI versus human-created jokes when there is uncertainty concerning their source—adhering to a heuristic that better jokes are typically a human’s and worse jokes are typically an AI’s. This supports findings that users favor the work of other humans over AI in subjective domains^10,11^. Further, this echoes previous findings wherein AI is only attributed responsibility or considered agentic when there is an unfavorable outcome^22^. However, Experiment 2 shows that such biases are absent when joke sources *are* provided, as reflected in similar ratings between human-created jokes framed as either human or AI-created. This suggests that if there are aversions toward AI in this domain, they are weak and easily overcome when presented with counterevidence (*i.e.*, good jokes framed as AI-created).

These results contribute to findings that user attitudes toward AI are malleable, even when supposed AI attempt feats believed the province of humans^7^. It is once again worth noting that the vast majority of participants in Experiment 2 believed our deception, implying a willingness to accept the ability of current AI systems to produce compelling, human-level jokes (when framed appropriately).

It would be interesting to further explore societal attitudes toward AI that perform other creative work, such as art or poetry. While AI have demonstrated degrees of proficiency in these realms, it remains unclear how they will be broadly received. For instance, findings suggest that the way we talk about AI—as a tool or agent—affects how users allocate credit for machine-generated artwork^23^. To understand human beliefs (and biases) regarding AI capability, it will be necessary to explore these domains.

There are a few limitations to our work. The first is that our findings are domain-specific by design. There are many factors believed to promote algorithm bias^5^. For example, there is evidence that experts are more likely to discount the quality of algorithmic advice than non-experts^13,24–26^. Future work may consider recruiting domain experts to assess potential differences in expressed bias (such as AI researchers and/or professional comedians, in the case of joke appraisal).

An influential finding is that aversion arises due to increased user sensitivity to AI mistakes—especially at the outset of tasks^8^. Users are more likely to overlook mistakes committed by themselves or another human compared to an algorithm. Since our jokes were randomly presented, we cannot assess if such order effects are present in our study (a “mistake” in this case being a poor-quality joke or non-joke). Subsequent work should evaluate potential order effects to see if this aversion is replicated.

Subsequent work may also categorize these jokes according to their content to see if it affects perceived funniness and source ascription. Indeed, people have shown differential ratings for computer-delivered jokes based on the appropriateness of their content^20^. Further, the admittedly impoverished nature of the present task (rating jokes on a computer screen) does not capture the many nuances and complexities of humorous communication. To further assess human receptivity toward and discriminability regarding AI humor, various other modes of humor and experimental designs may be used in future work (e.g., employing joking conversational agents)^27,28^. Such approaches can assess the external validity of this and related work.

Lastly, it should also be noted that the GPT-2 language model from which the actual AI-created jokes were taken has been succeeded by the GPT-3^29^, which was introduced after the completion of our study. The GPT-3 is reported to outperform its predecessor in naturalistic language, so using jokes from this newer model would be useful in assessing both the technical capability of and perceptions toward more advanced creative language systems.

## Conclusion

As AI systems advance, so do our attitudes toward them^30^. Indeed, our so-called “theory of machine”—our beliefs regarding what algorithms are capable of and how—seems to evolve with the technology itself^13^. More and more, AI challenge the delineation of human and machine—often beating us at our own game. This can be in critical tasks, such as making medical diagnoses, or in more casual pursuits, such as making jokes. Our results demonstrate that our beliefs may not be as fixed as once suggested and that we are open to AI occupying spaces once thought our own. Nonetheless, much work remains if we are to fully understand how and why biases toward AI occur.

## Methods

### Participants

Participants were recruited through Amazon Mechanical Turk. Forty-three participants were recruited for Experiment 1 and 83 participants were divided between two counterbalanced conditions in Experiment 2. Participant demographic information is reported in Supplementary Table S1. To be eligible for the our study, participants were required to meet the following criteria: (1) Have greater than or equal to 1000 Human Intelligence Tasks (HITs) approved; (2) Have greater than or equal to 98% HIT approval rate for all requesters’ HITs; (3) Be located in the United States; (4) Be fluent in English and; (5) Be 18-years-old or older.

All participants provided informed consent before taking part in our study. Furthermore, they were all debriefed about its true nature following its conclusion, including all deception. Following this, they once again consented to allow their data to be used in the final analysis. The University of California, Irvine Institutional Review Board approved this research, which was conducted according to its guidelines.

### Stimuli

Sixty items were adopted from a large database of jokes (*N* = 194,554) scraped from Reddit^31^. This set contains all jokes submitted to the subreddit r/jokes as of February 13, 2017. Jokes were curated by the first author to exclude those which were deemed potentially sexist, racist, or otherwise offensive. We also excluded jokes specific to a given time period or event (e.g., the 2016 US election) to avoid contextual dependencies.

To improve the feasibility of our cover story and provide a baseline for comparison, we inserted 20 actual, machine-produced jokes adopted from the subreddit r/SubSimulatorGPT2. These jokes were trained using a GPT-2 language model^32^ based on submissions in r/jokes.

A small collection of sample jokes is presented in Supplementary Table S2 (see: “Data Availability” for link to complete database of jokes).

### Procedure

At the start of the experiment, participants were directed to read the following text: “The purpose of this study is to test the quality of a new artificial intelligence (AI) joke engine, JOSH (“Joke Ontology and System of Humor”). JOSH is being developed by researchers and uses state-of-the-art deep learning algorithms to construct jokes. However, JOSH is still in the early stages of development and we need your feedback to gauge its effectiveness and to help identify ways to improve it. To do this, we would like you to rate jokes created by JOSH according to how funny you find them. Lastly, we are interested in evaluating how well JOSH’s jokes compare to jokes made by actual humans”. Here, the script diverged between experiments. For Experiment 1, participants were provided the following: “Following each joke, you will be asked to guess if it was created by JOSH (“AI”) or by a person (“HUMAN”)”. For Experiment 2, participants were provided the following: “Before each joke, you will be told if it was created by JOSH (“AI-GENERATED”) or by a person (“HUMAN-GENERATED”)”. This designation was in orange or blue boldface text above each joke. The jokes actually created by humans were alternatively framed as human or AI-created across two counterbalanced conditions, such that both framings were equally represented for each joke. The jokes actually created by AI were always accurately framed as AI-created.

Participants rated each randomly-presented joke based on its perceived funniness (0 = “not funny at all”; 5 = “very funny”). In Experiment 1, participants guessed each joke’s most likely source (“Definitely” or “Probably AI”; “Definitely” or “Probably Human”). Participants were debriefed regarding the true nature of our study, including the deception. They were then asked to indicate if they spotted our deception and, if so, to describe how and when they did to the best of their ability. Finally, they were asked whether they consented to their data being used in the final analysis.

### Analyses

Principal data analysis was conducted using JASP^33^. In addition to standard frequentist statistics, we also report Bayes factors (BFs). The advantages of using Bayesian inference, as well as suggested interpretations of results, are well-outlined in van Doorn et al.^34^.

## Supplementary Information

Supplementary Information.

## Data Availability

Stimuli used in and datasets generated during the current study are available through Open Science Framework: https://osf.io/bpt2d/?view_only=ec1fbeed317748d68ac3b4f170f1c7d9.
