# Supplementary material for: Perceptions of AI engaging in human expression
Source: Sci Rep. 2021 Oct 27;11:21181. doi: 10.1038/s41598-021-00426-z (PMC8551225; doi:10.1038/s41598-021-00426-z)
Supplement: Supplementary file 1 — Supplementary Information. [file 41598_2021_426_MOESM1_ESM.pdf]

Supplementary Table S1: Demographic characteristics of participants for both experiments.

Demographic Category	Exp. 1 N	Exp. 1 %	Exp. 2 N	Exp. 2 %
Gender				
Female	20	46.512	36	43.373
Male	23	53.488	46	55.422
Non-binary	-	-	1	1.205
Age				
21-29	6	13.953	6	7.229
30-39	16	37.209	30	36.145
40-49	12	27.907	23	27.711
50-59	8	18.605	16	19.277
60 or older	1	2.326	8	9.639
Race or ethnicity				
American Indian or Alaskan Native	1	2.326	-	-
Black, Afro-Caribbean, or African-American	3	6.977	6	7.229
East Asian or Asian American	4	9.302	6	7.229
Latino/a or Hispanic American	1	2.326	3	3.614
Middle Eastern or Arab American	1	2.326	1	1.205
Multiple races	1	2.326	2	2.410
Non-Hispanic White or Euro-American	32	74.419	65	78.313
Marital status				
Divorced	3	6.977	11	13.253
Married	16	37.209	31	37.349
Never married	23	53.488	38	45.783
Separated	1	2.326	-	-
Widowed	-	-	3	3.614
Employment status				
Disabled, not able to work	-	-	1	1.205
Employed, working 1-39 hours per week	9	20.930	22	26.506
Employed, working 40 or more hours per week	32	74.419	46	55.422
Not employed, NOT looking for work	1	2.326	6	7.229
Not employed, looking for work	-	-	3	3.614
Retired	1	2.326	5	6.024
Education				
Associate degree	5	11.628	7	8.434
Bachelor degree	13	30.233	33	39.759
Graduate degree	5	11.628	13	15.663
High school degree or equivalent (*e.g.*, GED)	5	11.628	10	12.048
Less than high school degree	-	-	1	1.205
Some college, but no degree	15	34.884	19	22.892
Annual household income				
$0-$29,000	6	13.954	23	27.711
$30,000-$59,000	26	60.465	24	28.646
$60,000-$89,000	7	16.279	23	27.710
$90,000 or more	4	9.303	13	15.663
Computer science/technology experience				
Has taken at least one college-level course in computer science	9	20.930	26	31.325
Has a computer science or engineering undergraduate degree	4	9.302	6	7.229
Has programming experience	6	13.953	11	13.253

Supplementary Table S2: Sample joke stimuli with their actual and most frequently guessed sources from Experiment 1.

Joke	Actual Source	Mode Guessed Source
My girlfriend told me to take the spider out instead of killing it. We went and had some drinks. Cool guy. Wants to be a web developer.	Human	Probably Human
As I suspected, someone has been adding soil to my garden. The plot thickens.	Human	Probably Human
I've recently developed a severe phobia of elevators. I'm taking steps to avoid them.	Human	Probably Human
The word 'nothing' is a palindrome. 'Nothing' reversed is 'Gnihton' which is also nothing.	Human	Probably AI
The bartender says "We don't serve time travelers in here." A time traveler walks into a bar.	Human	Probably AI
What's the stupidest animal in the jungle? The Polar bear.	Human	Probably AI
What do you get when you cross a hippie, a hipster, and a vegan? A... vegan? (I'll go with hippie to avoid the hipster joke.)	AI	Probably Human
How do you get a woman pregnant? Just say: "You too, baby."	AI	Probably AI
I was gonna tell the guy who invented the telephone how it works... But he's dead.	AI	Probably AI
Why won't the chicken fly over the balcony? Actually, you'll get it tomorrow.	AI	Probably AI
I tried to ask this guy out. It didn't go well.	AI	Definitely AI
Is it safe to eat in a restaurant while smoking? ... you'll get a bite on your bill.	AI	Definitely AI
When my dad came home he was surprised by the smell of bacon.	AI	Definitely AI

Note. There are no jokes for which the majority of participants guess the source to be "Definitely Human" and only one AI-created joke for which the majority of participants guess the source to be "Probably Human."
